# Prevalence and genotype distribution of caprine papillomavirus in peripheral blood of healthy goats in farms from three European countries

**DOI:** 10.3389/fvets.2023.1213150

**Published:** 2023-06-15

**Authors:** Anna Cutarelli, Francesca De Falco, Bianca Cuccaro, Vesna Milićević, Branislav Kureljušić, Jovan Bojkovski, Pellegrino Cerino, Antonella Perillo, Raluca Marica, Cornel Catoi, Sante Roperto

**Affiliations:** ^1^Istituto Zooprofilattico Sperimentale del Mezzogiorno, Portici, Italy; ^2^Dipartimento di Medicina Veterinaria e delle Produzioni Animali, Università degli Studi di Napoli Federico II, Napoli, Italy; ^3^Institute of Veterinary Medicine of Serbia, Belgrade, Serbia; ^4^Faculty of Veterinary Medicine, Department for Ruminants and Swine disease, Belgrade, Serbia; ^5^Dipartimento di Sanità pubblica, Università degli Studi di Napoli Federico II, Napoli, Italy; ^6^Medicina di Precisione e Rigenerativa e Area Jonica, Università degli Studi di Bari “Aldo Moro”, Bari, Italy; ^7^Pathology Department, Faculty of Veterinary Medicine, University of Agricultural Sciences and Veterinary Medicine, Cluj-Napoca, Romania

**Keywords:** blood, caprine papillomaviruses, ChPVs, droplet digital PCR, goat, liquid biopsy, molecular epidemiology

## Abstract

Caprine papillomaviruses (ChPVs, Capra hircus papillomaviruses) were detected and quantified for the first time using droplet digital polymerase chain reaction (ddPCR) *in* blood samples of 374 clinically healthy goats from farms located in Italy, Romania, and Serbia. Overall, ddPCR revealed ChPV DNA in 78 of the 374 examined samples, indicating that ~21% of the goats harbored circulating papillomavirus DNA. In particular, in Italian goat farms, ChPV genotypes were detected and quantified in 58 of 157 blood samples (~37%), 11 of 117 samples from Serbian farms (~9.4%), and 9 of 100 from Romanian blood samples (9%). Blood samples from Italian goat farms showed a high prevalence of ChPV1, which was detected in 45 samples (28.6%). The ChPV2 genotype was detected in 13 samples (~8.3%). Therefore, significant differences in prevalence and genotype distributions were observed. On Serbian and Romanian farms, no significant differences were observed in the genotype prevalence of ChPVs. Molecular findings are consistent with ChPV prevalence, characterized by a territorial distribution similar to that of papillomaviruses in other mammalian species. Furthermore, this study showed that ddPCR is a very sensitive and accurate assay for ChPV detection and quantification. The ddPCR may be the molecular diagnostic tool of choice, ultimately providing useful insights into the molecular epidemiology and field surveillance of ChPV.

## 1. Introduction

Papillomaviruses are a widespread family of pathogens that infect several mammalian and non-mammalian species (1). Overall, more than 400 papillomaviruses have been identified and sequenced including 50 genotypes of domestic ruminants (2). Papillomavirus infections have been observed in both large and small ruminants worldwide, and often have significant effects on livestock production (3). To date, 44 bovine papillomaviruses and four ovine papillomaviruses are known to cause infections in cattle and sheep, respectively (2). Bovine delta-papillomaviruses are highly pathogenic and are believed to be high-risk PV genotypes (4). Recent molecular surveys in large and small ruminants have been shown that there is a high prevalence of bovine and ovine papillomavirus infections in healthy cattle and sheep, respectively (5, 6). However, the extent of the caprine papillomavirus infection in goats remains unknown. Only two caprine papillomaviruses, *Capra hircus papillomavirus 1* (ChPV1), which belongs to the genus *Phipapillomavirus*, and ChPV2, which are currently unclassified, are known to occur in domestic goats (2). ChPV1 was isolated from the skin of a healthy 7-year-old goat (7), its genome of ChPV2 was obtained and characterized from teat lesions of a Damascus goat, and it appears to be closely related to *Xipapillomavirus1* species infecting cattle (8, 9).

Papillomavirus infection has been sporadically reported in goats (10), thereby very few cases of diseases associated with papillomavirus infection in goats have been documented. Although an infective agent has been proposed to be involved in goat papillomatosis of the mammary skin (11), the first papillomavirus-like sequences were reported in an outbreak of cutaneous fibropapillomas of the udder in Saanen goats (12). More recently, papillomavirus infection has been suggested to be a cause of caprine ocular squamous cell carcinoma (13). An immunohistochemical study performed on three goats with cutaneous papillomatosis suggested that benign neoplasia may be caused by papillomavirus infection (14).

This study aimed to investigate the prevalence and genotype distribution of ChPVs in whole blood of healthy goats from farms located in Italy, Romania, and Serbia, and to evaluate viral detection and load quantification using the droplet digital polymerase chain reaction (ddPCR) tool.

## 2. Materials and methods

### 2.1. Liquid biopsy samples and DNA extraction

Blood samples from 374 apparently healthy 1- to 3-year-old goats (157 samples were obtained from goats living in Italy, 117 from Serbia, and 100 from Romania) were collected from the jugular vein in vacutainers containing ethylenediaminetetraacetic acid at public and private slaughterhouses after permission of medical authorities. Furthermore, the official veterinarians responsible for the health conditions of the flocks, where the goats belonged, provided us with the medical records of the animals showing that the examined goats did not have any diseases or therapeutic treatment. Total DNA was extracted using a DNeasy Blood & Tissue Kit (Qiagen, Wilmington, DE, USA) according to the manufacturer's instructions.

### 2.2. Positive controls

Positive controls were artificially created by inserting 270 and 400 base pairs of the ChPV1 and ChPV2 E7 sequences, respectively, into a plasmid (vector: pUCIDT-AMP) (IDT, Integrated DNA Technologies, IA, USA).

### 2.3. Droplet digital polymerase chain reaction (ddPCR)

DNA extracted from 374 blood samples of healthy goats was analyzed using ddPCR. For ddPCR, the Bio-Rad QX100 ddPCR System was used according to the manufacturer's instructions as previously reported (15). The primer and probe sequences used to identify ChPV1 and ChPV2 are listed in Table 1. Finally, the results obtained by the ddPCR software QuantaSoft were converted directly into copy number/μL, multiplying the amount obtained by the instrument by 20 μL (the total volume of the reaction mixture) and then divided by 7 μL, that is, the volume of the DNA sample added at the beginning of the test. Each sample was analyzed in duplicate, and the samples were considered ChPV-positive if at least three droplets contained ChPV amplicons, as suggested for other papillomavirus infections (16, 17).

**Table 1:** Primers and probes used for the detection of circulating ChPV DNA through ddPCR.

	**Forward 5^′^3^′^**	**Reverse 5^′^3^′^**	**Probe (FAM)**	**Region**	**Size (bp)**	**Accession number**
ChPV1	AAACATTGTCTCCCGAGGTG	GTACAGCCTTAGTCCCCTATTG	TGCACAATGCCCCGCAATTTCA	E7	89	DQ091200.1
ChPV2	AGCCTACATTGCCTGACATC	ACAATTATAGCAGGCGACGG	AGAGGAATTAGAAGTTGAGGAGGCGGA	E7	137	MN148899.1

### 2.4. Statistical analysis

McNemar's test for two Related Binomial Proportions was used to evaluate the agreement between two tests performed on the same animals. The agreement between the two tests performed on different groups of animals from Italy, Romania, and Serbia (Italy vs. Romania and Italy vs. Serbia) was investigated using the Fisher's test. Statistical significance was set at *P* < 0.05. Statistical analyses were performed using R Studio.

## 3. Results

**Figure 1:**
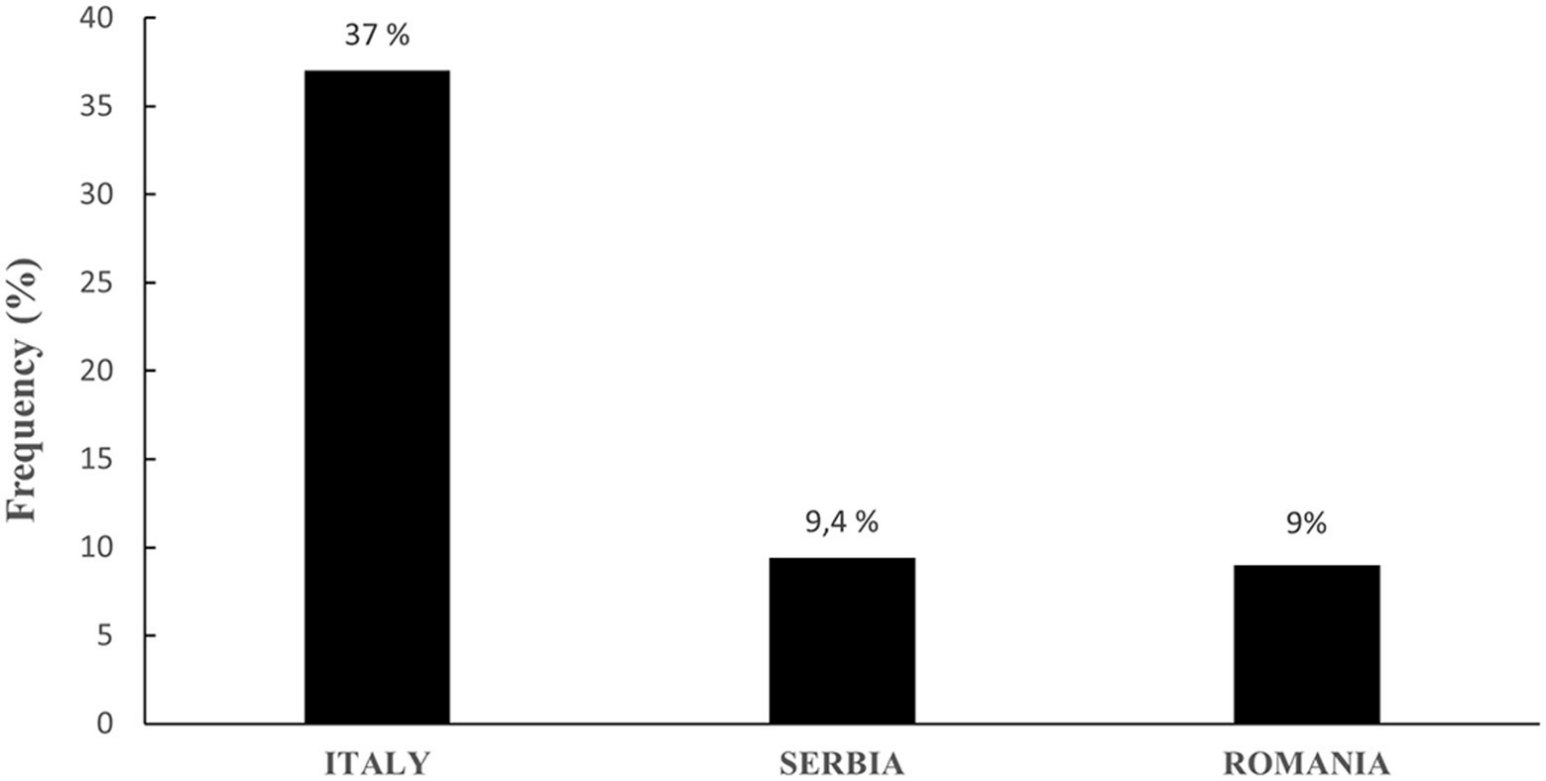
Overall prevalence of ChPVs in Italian, Serbian, and Romanian goat farms.

**Figure 2:**
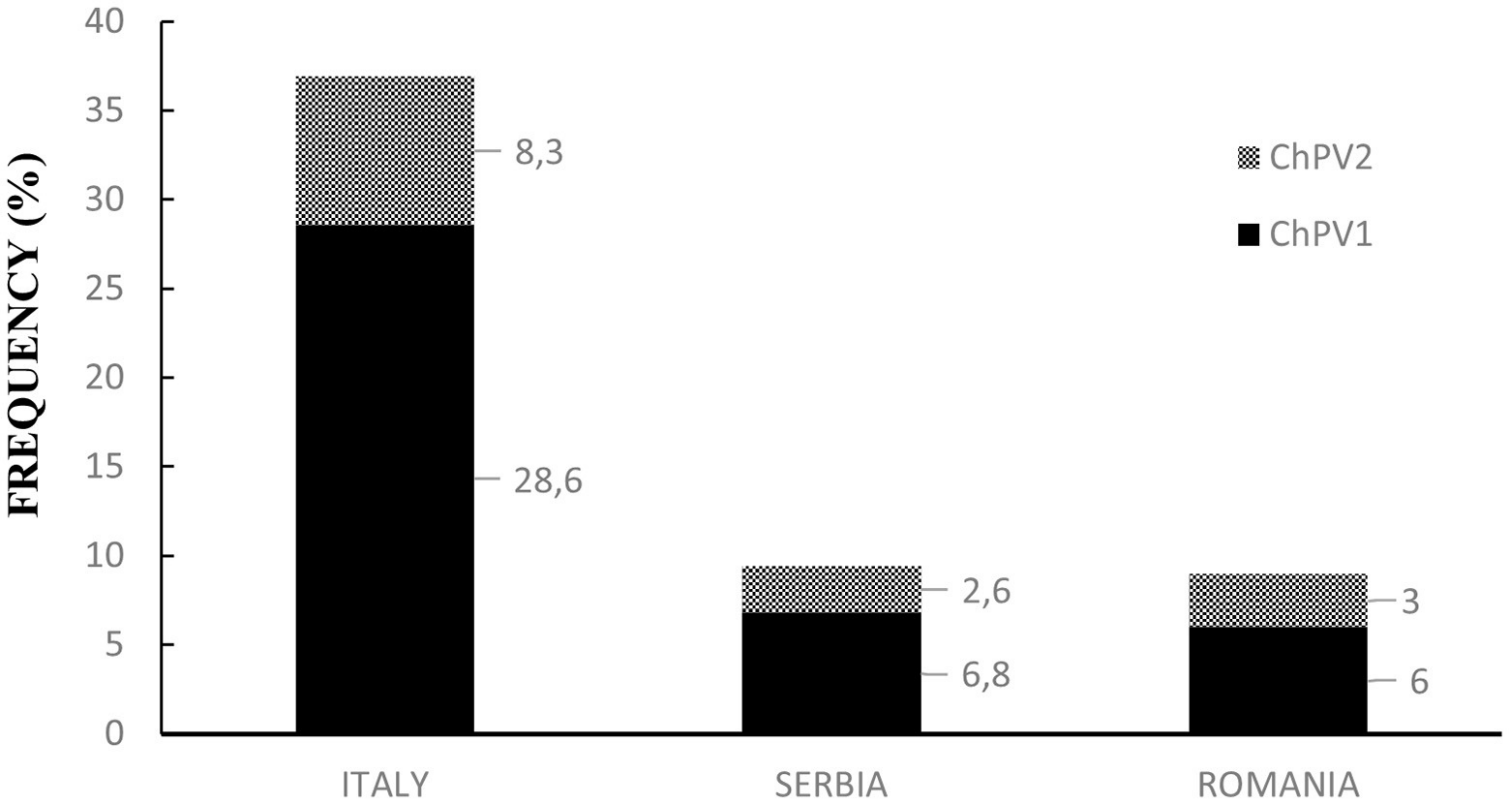
Prevalence and genotype distribution of ChPVs in Italian, Serbian, and Romanian goat farms.

Overall, ddPCR revealed ChPV DNA in 78 of the 374 examined blood samples, indicating that ~21% of the goats harbored circulating papillomavirus DNA (Supplementary Table 1). Rain plots of ddPCR for ChPV DNA are showed in Supplementary Figure 1. In particular, in Italian goat farms, ChPV genotypes were detected and quantified in 58 of 157 blood samples (~37%), 11 of 117 samples from Serbian farms (~9.4%), and 9 of 100 from Romanian blood samples (9%). The results are shown in Figure 1. Blood samples from Italian goat farms showed a high prevalence of ChPV1, which was detected in 45 samples (28.6%). The ChPV2 genotype was detected in 13 samples (~8.3%). McNemar's test showed significant differences in the prevalence of the two caprine genotypes (*P* < 0.05). In 117 samples from Serbian goat farms, eight samples were positive for ChPV1 and three for ChPV2, which were 6.8 and 2.6%, respectively. ChPV1 was observed in six of the 100 (6%) samples from Romania, whereas ChPV2 was detected in only three (3%) samples. On both Serbian and Romanian farms, no significant differences were observed in the genotype prevalence of ChPVs. Significant differences in both prevalence and genotype distribution were observed between Italian farms and Serbian and Romanian goat farms. Figure 2 shows the results for all the goat farms.

## 4. Discussion

Only isolated and sporadic ChPV DNA associated with neoplastic lesions has been reported in goats associated with neoplastic lesions (10). Therefore, the ecological epidemiology of ChPV genotypes remains unknown. To the best of our knowledge, this is the first systematic survey of the prevalence and genotype distribution of ChPVs among healthy goats from several farms in three European countries using ddPCR as a diagnostic assay, which has not yet been utilized for studying ChPV epidemiology. The current study shows that ddPCR is an advanced technology that can accurately diagnose ChPV infection with high specificity and sensitivity, thus representing a promising new tool for the accurate detection and quantification of ChPV nucleic acid load. This diagnostic approach quantifies a very small amount (< 1 copy number/μL) of ChPV DNA, thus making it an important diagnostic procedure capable of detecting otherwise undetectable viral DNA 500 times (maximum) more sensitive than qPCR for low-level analyte (18). Notably, ddPCR has been shown to be the most accurate and sensitive method for quantifying the nucleic acids of bovine and ovine papillomaviruses in cattle and sheep, respectively (5, 6, 19).

On Italian goat farms, a high prevalence of ChPV1 and ChPV2 was detected in healthy goats, with the ChPV1 genotype being the most prevalent. There were no significant differences in the prevalence of ChPV1 and ChPV2 genotypes between Romanian and Serbian goat farms. Overall, both ChPV genotypes showed a significant geographical distribution, with a significantly higher prevalence in Italian goat farms than in Serbian and Romanian farms. We cannot exclude the fact that these data may be attributable to the limited number of blood samples examined from these countries.

As ChPVs have been found in healthy goats, it is conceivable that the peripheral blood represents an important primary route of infection and that ChPVs may spread through the bloodstream. However, the role of ChPVs in the pathogenesis of viral diseases in goats remains unknown. ChPV can cause persistent host infections, posing a threat to the goat industry. Therefore, new experimental therapeutic protocols against ChPV infections are being developed (20).

Finally, ddPCR can be used to improve diagnostic methods to allow researchers to accurately identify the genotypic distribution of ChPVs and better understand the territoriality of ChPV circulation. Gaining insight into the territorial prevalence and genotype distribution of ChPVs is important for improving the molecular and ecological epidemiology of ChPVs, as papillomavirus genotypes may be related to specific diseases, as has been reported in other animal species. Furthermore, the identification of the natural reservoirs of infectious pathogens, including viruses, plays a crucial role in controlling disease outbreaks in domestic animals, especially those diseases for which neither immunological nor therapeutic prophylaxis exists.

## Data availability statement

The original contributions presented in the study are included in the article/Supplementary material, further inquiries can be directed to the corresponding author.

## Ethics statement

In this study, animal experiments were not performed. All the samples were collected from slaughterhouses, and therefore, no ethical approval was required.

## Author contributions

SR, CC, and BK designed the experiments. AC, FD, BC, and VM carried out the experiments. SR, PC, AP, JB, and RM analyzed data. SR wrote the manuscript. All authors have read and approved the final manuscript.

## References

[1] Shah SD Doorbar J Goldstein RA. Analysis of host-parasite incongruence in Papillomavirus evolution using importance sampling. Mol Biol Evol. (2010) 27:1301–14. 10.1093/molbev/msq015 20093429 PMC2872622

[2] The Papillomavirus Episteme. (2023). Available online at: pave.niaid.nih.gov/ (accessed June 3, 2023).

[3] Medeiros-Fonseca B Abreu-Silva AL Medeiros R Oliveira PA Gil da Costa RM. *Pteridium spp*. and bovine papillomavirus: partners and cancer. Front Vet Sci. (2021) 8:758720. 10.3389/fvets.2021.758720 34796228 PMC8593235

[4] Daudt C Da Silva FRC Lunardi M Alves CBDT Weber MN Cibulski SP. Papillomaviruses in ruminants: an update. Transbound Emerg Dis. (2018) 65:1381–95. 10.1111/tbed.12868 29603890

[5] De Falco F Corrado F Cutarelli A Leonardi L Roperto S. Digital droplet PCR for the detection and quantification of circulating bovine Deltapapillomavirus. Transbound Emerg Dis. (2021) 68:1345–52. 10.1111/tbed.13795 33350088

[6] De Falco F Cutarelli A D'Alessio N Cerino P Catoi C Roperto S. Molecular epidemiology of ovine papillomavirus infections among sheep in southern Italy. Front Vet Sci. (2021) 8:790392. 10.3389/fvets.2021.790392 34881323 PMC8645557

[7] Van Doorslaer K Rector A Vos P Van Ranst M. Genetic characterization of the Capra hircus papillomavirus: a novel close-to-root artiodactyl papillomavirus. Virus Res. (2006) 118:164–9. 10.1016/j.virusres.2005.12.007 16430985

[8] Dogan F Dorttas SD Dagalp SB Ataseven VS Alkan F. A teat papillomatosis case in a Damascus goat (Shami goat) in Hatay Province, Turkey: a new putative papillomavirus? Arch Virol. (2018) 163:1635–42. 10.1007/s00705-018-3781-2 29502149

[9] Willemsen A van der Boom A Dietz J Dagalp SB Dogan F Bravo IC. Genomic and phylogenetic characterization of ChPV2, a novel goat closely related to the Xi-PV1 species infecting bovines. Virol J. (2020) 17:167. 10.1186/s12985-020-01440-9 33126890 PMC7602357

[10] Odhah MNA Garba B Wen CX Reduan MFH. A rare case of oral papillomatosis in a goat kid. Case Rep Vet Med. (2022) 2022:1598256. 10.1155/2022/1598256 35571504 PMC9098364

[11] Theilen G Wheeldon EB East N Madewell B Lancaster WB Munn R. Goat papillomatosis. Am J Vet Res. (1985) 46:2519–26. 3002215

[12] Manni V Roperto F Di Guardo G Galati D Condoleo R Venuti A. Presence of papillomavirus-like DNA sequences in cutaneous fibropapillomas of the goat udder. Vet Microbiol. (1998) 61:1–6. 10.1016/S0378-1135(98)00168-0 9646460

[13] Marà M Di Guardo G Venuti A Marruchella G Palmieri C De Rugeriis M. Spontaneous ocular squamous cell carcinoma in twin goats: pathological and biomolecular studies. J Comp Pathol. (2005) 132:96–100. 10.1016/j.jcpa.2004.06.007 15629484

[14] Al-Salihi KA Al-Dabhawi AA Ajeel AA Erzuki IA and Ali TAH. (2020). Clinico-histopathological and immunohistochemical study of ruminant's cutaneous papillomavirus in *Iraq*. Vet. Med. Int. 2020, 5691974, 10.1155/2020/5691974 32148749 PMC7054784

[15] Cutarelli A De Falco F Uleri V Buonavoglia C Roperto S. The diagnostic value of the droplet digital PCR for the detection of bovine deltapapillomavirus in goats by liquid biopsy. Transbound Emerg Dis. (2021) 68:3624–30. 10.1111/tbed.13971 33386672

[16] Jeannot E Latouche A Bonneau C Calméjane MA Beaufort C Ruigrok-Ritstier K. Circulating HPV DNA as a marker for early detection of relapse in patients with cervical cancer. Clin Cancer Res. (2021) 27:5869–77. 10.1158/1078-0432.CCR-21-0625 34210686 PMC9401545

[17] De Falco F Cuccaro B De Tullio R Alberti A Cutarelli A De Carlo E. (2023). Possible etiological association of ovine papillomaviruses with bladder tumors in cattle. Virus Res. 328, 199084. 10.1016/j.virusres.2023.199084 36878382 PMC10194246

[18] Suo T Liu X Feng J Gun M Guo D and Ullah H. (2020). ddPCR: a more accurate tool for SARS-CoV-2 detection in low viral load specimens. Emerg. Microbes Infect. 9, 1259–1268. 10.1080/22221751.2020.1772678 32438868 PMC7448897

[19] Roperto S Cutarelli A Corrado F De Falco F and Buonavoglia C. (2021). Detection and quantification of bovine papillomavirus DNA by digital droplet PCR in sheep blood. Sci. Rep. 11, 10292. 10.1038/s41598-021-89782-4 33986444 PMC8119674

[20] Bozkurt G Kaya F Yildirim Y Yildiz R Gungor O Dogan F. The effect of multiple-dose ivermectin treatment on CD4^+^/CD8^+^ and the oxidative stress index in goats with udder viral papillomatosis. Res Vet Sci. (2023) 157:17–25. 10.1016/j.rvsc.2023.02.006 36848794

