# Supplementary material for: Prevalence and genotype distribution of caprine papillomavirus in peripheral blood of healthy goats in farms from three European countries
Source: Front Vet Sci. 2023 Jun 15;10:1213150. doi: 10.3389/fvets.2023.1213150 (PMC10310300; doi:10.3389/fvets.2023.1213150)
Supplement: Supplementary file 1 [file Data_Sheet_1.ZIP › caption supplemental Figure S1.docx]

Supplemental Figure S1. Rain plots of ddPCR for ChPV1 (A) and ChPV2 (B) DNA detection. Blue droplets = droplets positive; gray droplets = droplets negative for ChPV DNA.
