# Supplementary material for: Prevalence and genotype distribution of caprine papillomavirus in peripheral blood of healthy goats in farms from three European countries
Source: Front Vet Sci. 2023 Jun 15;10:1213150. doi: 10.3389/fvets.2023.1213150 (PMC10310300; doi:10.3389/fvets.2023.1213150)
Supplement: Supplementary file 1 [file Data_Sheet_1.ZIP › table S1.docx]

| **COUNTRY** | **ChPV1** | **ChPV2** | **ChPV1** | **ChPV2** | **ChPV1** | **ChPV2** | **ChPV1** | **ChPV2** | **ChPV1** | **ChPV2** | **ChPV1** | **ChPV2** |
| --- | --- | --- | --- | --- | --- | --- | --- | --- | --- | --- | --- | --- |
| **ITALY**  **Total: 157**  **ChPV1: 45**  **ChPV2:13** | N | N | N | 0.6 | N | N | N | N | N | N | N | N |
|  | 0.419 | N | N | N | 0.209 | N | N | N | N | N | N | N |
|  | N | N | 1.3 | N | N | N | 1.09 | N | N | N | N | N |
|  | N | N | 1.3 | N | 0.14 | N | 0.8 | N | N | N | N | N |
|  | 1.64 | N | N | N | N | N | N | N | N | N | N | N |
|  | N | N | 0.97 | N | N | 0,5 | 0.12 | N | N | N | N | N |
|  | 2.25 | N | 0.7 | N | 2.03 | N | 1.6 | N | N | N | N | N |
|  | N | N | N | N | N | N | N | N | N | N |  |  |
|  | 0.365 | N | 0.6 | N | N | N | N | N | N | N |  |  |
|  | 0.8 | N | 0.4 | N | N | N | 1.11 | N | N | N |  |  |
|  | N | N | 0.25 | N | N | N | 1.12 | N | N | N |  |  |
|  | 0.447 | N | 0.6 | N | N | N | N | N | 0.7 | N |  |  |
|  | N | N | N | N | N | N | N | N | 1.7 | N |  |  |
|  | N | N | N | N | N | N | N | N | N | N |  |  |
|  | N | N | N | N | N | N | N | N | N | N |  |  |
|  | N | N | 10 | 2.94 | 0.56 | 0.77 | N | N | N | N |  |  |
|  | N | N | 1.45 | N | N | 0.92 | N | 0.6 | N | N |  |  |
|  | N | N | 0.83 | N | 0,25 | N | N | N | N | N |  |  |
|  | N | N | N | N | N | N | N | N | N | N |  |  |
|  | 2.2 | N | N | N | 1.14 | 0.54 | N | 0.31 | N | N |  |  |
|  | N | N | N | N | N | N | N | N | N | N |  |  |
|  | 1.3 | N | N | N | N | N | 0.32 | N | N | N |  |  |
|  | N | N | 1.3 | N | N | N | 1.4 | 0.34 | N | N |  |  |
|  | 0.6 | 1.6 | 1.25 | N | N | N | N | 0.3 | 0.7 | N |  |  |
|  | N | N | N | N | N | N | N | N | N | N |  |  |
|  | N | N | 0.7 | N | 1.4 | N | N | N | N | N |  |  |
|  | N | N | N | N | 1.9 | 0.562 | N | N | N | N |  |  |
|  | N | N | N | N | N | N | N | N | N | N |  |  |
|  | 0.9 | N | N | N | 0.5 | N | N | N | N | N |  |  |
|  | 1.7 | N | N | N | N | 0.7 | N | N | N | N |  |  |

| **COUNTRY** | **ChPV1** | **ChPV2** | **ChPV1** | **ChPV2** | **ChPV1** | **ChPV2** | **ChPV1** | **ChPV2** |
| --- | --- | --- | --- | --- | --- | --- | --- | --- |
| **ROMANIA**  **TOTAL: 100**  **ChPV1: 6**  **ChPV2: 3** | N | N | N | N | N | N | N | N |
|  | N | N | N | N | N | N | 0.513 | N |
|  | N | N | N | N | N | N | N | N |
|  | N | N | N | N | N | N | 0.185 | 0.8 |
|  | N | N | N | N | N | N | N | N |
|  | N | N | N | N | N | N | N | N |
|  | N | N | N | N | 0.704 | N | N | N |
|  | N | N | N | N | N | N | N | N |
|  | N | N | N | N | N | N | N | N |
|  | N | N | N | N | N | N | N | N |
|  | N | N | N | N | N | N |  |  |
|  | N | N | N | N | N | N |  |  |
|  | N | N | 0.646 | N | N | N |  |  |
|  | N | N | N | N | N | N |  |  |
|  | N | 0.159 | N | N | N | N |  |  |
|  | N | N | N | N | N | N |  |  |
|  | N | N | N | N | N | N |  |  |
|  | N | N | N | N | 0.65 | N |  |  |
|  | N | N | N | N | N | N |  |  |
|  | N | N | N | N | N | N |  |  |
|  | N | N | N | N | N | 0.48 |  |  |
|  | N | N | N | N | N | N |  |  |
|  | N | N | N | N | N | N |  |  |
|  | N | N | N | N | N | N |  |  |
|  | N | N | N | N | N | N |  |  |
|  | N | N | N | N | N | N |  |  |
|  | N | N | N | N | 0.0852 | N |  |  |
|  | N | N | N | N | N | N |  |  |
|  | N | N | N | N | N | N |  |  |
|  | N | N | N | N | N | N |  |  |

| **COUNTRY** | **ChPV1** | **ChPV2** | **ChPV1** | **ChPV2** | **ChPV1** | **ChPV2** | **ChPV1** | **ChPV2** |
| --- | --- | --- | --- | --- | --- | --- | --- | --- |
| **SERBIA**  **TOTAL: 117**  **ChPV1: 8**  **ChPV2: 3** | N | N | N | N | N | N | N | N |
|  | N | N | N | N | N | N | N | N |
|  | N | N | N | N | N | 1 | N | N |
|  | 1.23 | N | N | N | N | N | 1 | N |
|  | 0.85 | N | N | N | N | N | N | N |
|  | N | N | N | N | N | N | N | N |
|  | 0.887 | N | N | N | N | N | N | N |
|  | N | N | N | N | 0.715 | N | N | N |
|  | N | N | N | N | N | N | N | N |
|  | N | N | N | N | N | N | 0.9 | N |
|  | N | N | N | N | N | N | N | N |
|  | N | N | N | N | N | N | N | N |
|  | N | N | 0.288 | N | N | N | N | N |
|  | N | N | N | N | N | N | N | N |
|  | N | N | N | N | N | N | N | N |
|  | N | N | N | N | N | N | 0.8 | N |
|  | N | N | N | N | N | N | N | N |
|  | N | N | N | N | N | N | N | N |
|  | N | N | N | N | N | N | N | N |
|  | N | N | N | N | N | N | N | N |
|  | N | N | N | N | N | N | N | N |
|  | N | N | N | N | N | N | N | N |
|  | N | N | N | N | N | N | N | N |
|  | N | N | N | N | N | N | N | N |
|  | N | N | N | N | N | N | N | N |
|  | N | N | N | N | N | 1.2 | N | N |
|  | N | N | N | N | N | N | N | N |
|  | N | N | N | N | N | N |  |  |
|  | N | N | N | N | N | N |  |  |
|  | N | 0.635 | N | N | N | N |  |  |

Table S1 shows the overall detection and quantification of ChPVs in blood samples from Italian, Romanian, and Serbian goat farms.
