# Supplementary figures and images for: Prevalence and genotype distribution of caprine papillomavirus in peripheral blood of healthy goats in farms from three European countries
Source: Front Vet Sci. 2023 Jun 15;10:1213150. doi: 10.3389/fvets.2023.1213150 (PMC10310300; doi:10.3389/fvets.2023.1213150)

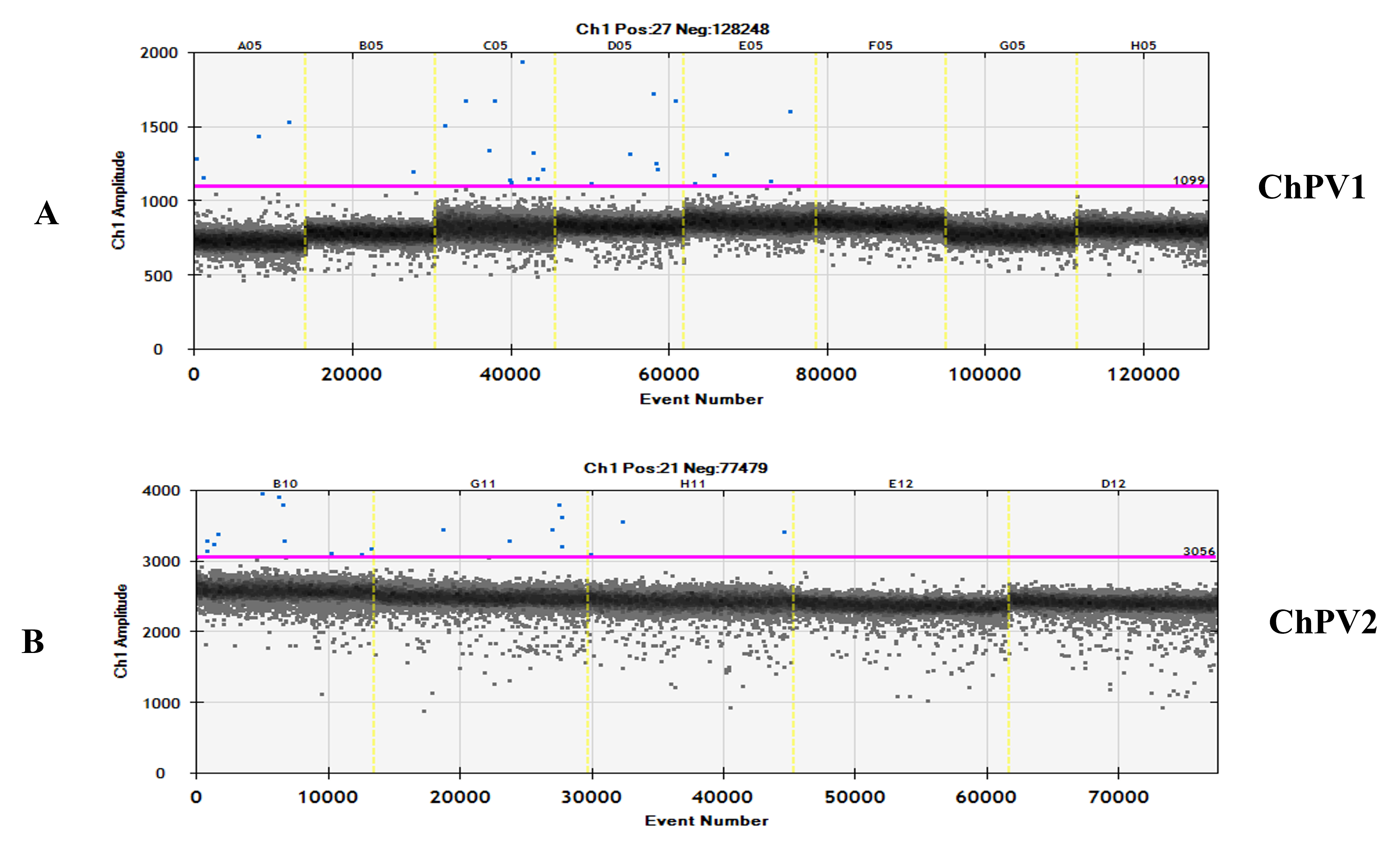

Supplement: Supplementary file 1 [file Data_Sheet_1.ZIP › S1.tiff]
